# Reply: Missing ligand model in autologous stem cell transplantation

**DOI:** 10.1038/sj.bjc.6604154

**Published:** 2007-12-18

**Authors:** W Leung, R Handgretinger, V Turner, G A Hale

**Affiliations:** 1 Department of Oncology, St Jude Children's Research Hospital, Memphis, TN, USA; 2 Department of Pediatrics, University of Tennessee Health Science Center, Memphis, TN, USA; 3 Department of Pathology, St Jude Children's Research Hospital, Memphis, TN, USA

**Sir**,

We agree with Stern *et al* that missing KIR-ligand does not imply inhibitory KIR–HLA receptor–ligand mismatch. In fact, 2 out of our 16 patients (13%) would have been misclassified using the analytic approach of Stern *et al* (one for KIR3DL1–Bw6/6 and the other for KIR2DL1–HLA-C^Asn80/Asn80^). The statistical implication for misclassification and exclusion of patients missing Bw4 cannot be overemphasised. For example, previous publication studying the effects of HLA mismatch has already demonstrated the statistical effect (bias towards null) with misclassification involving only 2–3 patients among 130 individuals (Leung *et al*, 2001). Furthermore, exclusion of KIR3DL1–Bw4 from any examination of KIR–HLA interaction will have similar statistical effect. For instance in our study, a prominent KIR3DL1 mismatch effect was observed: six out of the seven patients (86%) with mismatch involving KIR3DL1 were alive, whereas only two out of the nine patients (22%) without KIR3DL1 mismatch were alive (*P*=0.01, Figure 1). If the analytic approaches of Stern *et al* were used on our cohort, we would have concluded erroneously that neither inhibitory KIR–HLA receptor–ligand mismatch nor the number of mismatch pairs had any effect on clinical outcome (HR for relapse=0.49 (95% CI: 0.1–2.8), *P*=0.43). Therefore, we agree with Stern *et al* that our intriguing findings should be confirmed in large studies, which use biologically appropriate model, prospective design, uniform conditioning regimen and graft processing, analyses accounting for confounders such as disease type or status, and direct assessment of KIR expression on NK cells providing proof that all patients who are assumed to possess and express all relevant inhibitory KIRs indeed do so (Leung *et al*, 2004, 2005).

## Figures and Tables

**Figure 1:**
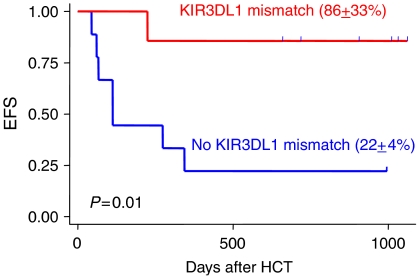
Event-free survival (EFS) of patients with receptor–ligand mismatch involving KIR3DL1 and of those with no mismatch.
